# Pfaffosidic Fraction from *Hebanthe paniculata* Induces Cell Cycle Arrest and Caspase-3-Induced Apoptosis in HepG2 Cells

**DOI:** 10.1155/2015/835796

**Published:** 2015-05-13

**Authors:** Tereza Cristina da Silva, Bruno Cogliati, Andréia Oliveira Latorre, Gokithi Akisue, Márcia Kazumi Nagamine, Mitsue Haraguchi, Daiane Hansen, Daniel Soares Sanches, Maria Lúcia Zaidan Dagli

**Affiliations:** ^1^Department of Pathology, School of Veterinary Medicine and Animal Science, University of São Paulo, Avenida Prof. Dr. Orlando Marques de Paiva 87, Cidade Universitária, 05508-900 São Paulo, SP, Brazil; ^2^School of Pindamonhangaba, Avenida Nossa Senhora do Bom Sucesso 3344, Campo Alegre, 12420-010 Pindamonhangaba, SP, Brazil; ^3^Section of Pharmacology, Division of Animal Biology, Biological Institute of São Paulo, Avenida Conselheiro Rodrigues Alves 1.252, Vila Mariana, 04014-002 São Paulo, SP, Brazil

## Abstract

*Hebanthe paniculata* roots (formerly* Pfaffia paniculata* and popularly known as Brazilian ginseng) show antineoplastic, chemopreventive, and antiproliferative properties. Functional properties of these roots and their extracts are usually attributed to the pfaffosidic fraction, which is composed mainly by pfaffosides A–F. However, the therapeutic potential of this fraction in cancer cells is not yet entirely understood. This study aimed to analyze the antitumoral effects of the purified pfaffosidic fraction or saponinic fraction on the human hepatocellular carcinoma HepG2 cell line. Cellular viability, proliferation, and apoptosis were evaluated, respectively, by MTT assay, BrdU incorporation, activated caspase-3 immunocytochemistry, and DNA fragmentation assay. Cell cycle was analyzed by flow cytometry and the cell cycle-related proteins were analyzed by quantitative PCR and Western blot. The cells exposed to pfaffosidic fraction had reduced viability and cellular growth, induced G2/M at 48 h or S at 72 h arrest, and increased sub-G1 cell population via cyclin E downregulation, p27^KIP1^ overexpression, and caspase-3-induced apoptosis, without affecting the DNA integrity. Antitumoral effects of pfaffosidic fraction from* H. paniculata* in HepG2 cells originated by multimechanisms of action might be associated with cell cycle arrest in the S phase, by CDK2 and cyclin E downregulation and p27^KIP1^ overexpression, besides induction of apoptosis through caspase-3 activation.

## 1. Introduction

Hepatocellular carcinoma (HCC) is known as the 5th most common human cancer in the world; and it has the third highest mortality rate among all cancers [1]. Despite major efforts to improve treatment of HCC, therapeutic options remain limited. Therapies with pharmacological agents or alternative strategies fail to substantially improve the prognosis of patients with unresectable HCC [2]. Therefore, it is necessary to discover novel agents that can be used as adjuvant for HCC therapy [3]. In this manner,* Hebanthe paniculata* a plant traditionally used in Brazilian folk medicine has drawn attention to its antineoplastic effects on several animal and cell models [4–8].

*Hebanthe paniculata* (formerly known as* Pfaffia paniculata*) belongs to the Amaranthaceae family and is popularlyknown as Brazilian ginseng. Its main components are stigmasterol, sitosterol, allantoin, pfaffic acid, and glycosides, which are triterpenoid saponins, denominated pfaffosides A, B, C, D, E, and F [9]. These saponins are considered the main active components of* H. paniculata* roots and have several biological properties described [10].

Previous studies from our group showed antineoplastic effects of* H. paniculata* roots and its extracts on several animal and cell models [4–8]. Gavage with powdered roots showed growth inhibitory effects in Ehrlich tumor-bearing mice [4]. Likewise, mice submitted to hepatocarcinogenesis model and fed with powdered roots showed reduction in incidence, mean area, and number of liver preneoplastic lesions [5]; also, it was observed to have decreased cell proliferation and induction of apoptosis, suggesting its chemopreventive activity [6]. In addition, the butanolic extract of* H. paniculata* roots increased survival in mice with the ascitic form of Ehrlich tumor [7] and showed antiproliferative effects in human mammary adenocarcinoma cells [8]. These findings suggest the presence of antineoplastic compounds in butanolic extract, like pfaffosides A–F.

Studies with purified pfaffosides showed inhibitory effects on the growth of cultured murine melanoma B-16 cells [9, 11]. However, antitumoral effects of pfaffosidic fraction from* H. paniculata* roots have been poorly examined. Thus, to obtain insights into its mechanism of action, we used the human HCC cell line HepG2 and examined the effects of purified pfaffosidic fraction on survival, on cell cycle distribution, on apoptosis, and on the levels of expression of several cell cycle control proteins.

## 2. Materials and Methods

### 2.1. Cell Culture

Human HCC cell line (HepG2) was kindly provided by Dr. Ana Paula de Melo Loureiro (School of Pharmaceutical Sciences, University of São Paulo, São Paulo, Brazil). Cells were cultured in DMEM (Gibco, Invitrogen) with HEPES (10 mM) and supplemented with 10% fetal bovine serum. Cells were maintained at 37°C in a humidified atmosphere with 5% CO_2_. All treatments with pfaffosidic fraction were started after 24 h of cell culture.

### 2.2. Plant Material

*H. paniculata* roots were kindly provided by Dr. Gokithi Akisue. A specimen of this plant was confirmed by the identification of its characteristic flowers and leaves deposited in the Goro Hashimoto (São Paulo, Brazil) Herbarium (n° 37,411).

### 2.3. Extraction of Pfaffosidic Fraction (SF-100%)

The extract was prepared as previous described [12]. Briefly,* H. paniculata* powdered roots were extracted with ethanol (EtOH). The EtOH extract was concentrated under reduced pressure, at 55°C, in rotary evaporator to provide the crude residue. The residue obtained was dried in a desiccator until reaching a constant weight. The butanolic extract was obtained by partition of ethanolic extract in n-butanol-water mixture. The butanolic phase was evaporated by reduced-pressure evaporation and eluted with increasing gradients of methanol : water of 0 (0 : 100; 20 : 80; 40 : 60; 60 : 40; 80 : 20 and 100 : 0) up to 100% methanol (MeOH). This procedure provided five pfaffosidic fractions according to MeOH solubility: 20, 40, 60, 80, and 100%. In previous experiments, we demonstrated that the 100% fraction (SF-100%) showed greater cell growth inhibition in comparison with other fractions (data not shown). Thus, we analyzed only the antitumoral effects of SF-100% in this study. Solvent was evaporated and the fraction was dried and stored at −20°C until use.

### 2.4. Pfaffosidic Fraction Treatment

Before assays, the dried fraction was dissolved in tween-20 (1 L/mg extract; Sigma Aldrich), diluted in phosphate-buffered saline (PBS), and sterilized with 0.22 *μ*m pore size filter (Millipore). The final work dilution (100 *μ*g/mL) was performed in culture medium DMEM with 10% FBS (Gibco, Invitrogen). HepG2 cells were plated with different concentrations according to the experimental analysis. In all procedures, cells were incubated with the SF-100% (100 *μ*g/mL) for 24, 48, and 72 h (*n* = 6 wells/time). In the control group, cells were incubated in the same conditions, without the pfaffosidic fraction.

### 2.5. Cell Cytotoxicity Assay (MTT Assay)

1 × 10^4^ cells/well were plated on 96-well plates and underwent SF-100% treatment for 24, 48, and 72 h. After each treatment time, cells were cultured with an MTT (3-(4.5-dimethylthiazol-2-yl)-2.5-diphenyltetrazolium bromide, Amresco, Inc., EUA) at 5 mg/mL, for 3 h. Then, the culture medium was removed, 100 mL DMSO was added, and the well absorbance was determined at a wavelength of 570 nm.

### 2.6. Cell Cycle Analysis by Flow Cytometry

2 × 10^6^ cells/well were plated on 6-well plates and underwent SF-100% treatment for 24, 48, and 72 h. Cell cycle analysis was performed as previously described [13]. Briefly, cells were harvested in PBS, fixed in cold 70% ethanol, and stored at −20°C until use. The ethanol was removed after washing three times in PBS (1800 rpm, 10 min). The pellet was resuspended in 200 *μ*L of propidium iodide (PI, Sigma Aldrich) solution (20 *μ*g/mL PI, 200 *μ*g/mL RNase A and 0.1% triton v/v in PBS) and incubated for 15 min at 37°C in absence of light. After the incubation, 10000 events were acquired by flow cytometry using a Becton Dickinson (FACScan Becton Dickinson Immunocytometry System) laser-based flow cytometry. Samples were analyzed by the FlowJo 7.2.2 software (Tree Star Inc., Ashland, OR, USA) and the results displayed as percentage of cells in sub-G1, G0/G1, S, and G2/M phases.

### 2.7. BrdU Incorporation

Cells in S phase of cell cycle were identified by the incorporation of 5-bromo-2′-deoxyuridine (BrdU, Sigma Aldrich) followed by immunocytochemical detection. Initially, a sterile stock solution of 0.1 M BrdU in PBS was prepared and stored in the dark at −20°C until use. 2.5 × 10^5^ cells/well were plated on 4-well cell culture slides (BD Falcon) and underwent SF-100% treatment for 24, 48, and 72 h. One hour before the end of each treatment period, 10 *μ*L of the BrdU stock solution was added in each well (final concentration of 100 *μ*M). Cells were fixed in buffered-paraformaldehyde (4%, pH 7.4) at 4°C for 10 min to the immunocytochemical detection of BrdU-positive cells.

### 2.8. Immunocytochemistry for BrdU and Activated Caspase-3

For immunocytochemical analysis, 2.5 × 10^5^ cells/well were plated on 4-well cell culture slides (BD Falcon). With exception of caspase-3 staining, cells were first permeabilized with acetone for 10 min at −20°C. Endogenous peroxidase was blocked by immersion in 5% H_2_O_2_ solution in methanol for 30 min and nonspecific binding sites were blocked with 5% skim milk for 30 min. Then, cell culture slides were incubated with primary antibodies monoclonal mouse anti-BrdU (1 : 100 in DNase-I solution, GE Healthcare) for one hour at RT or monoclonal mouse anti-cleaved caspase-3 (1 : 100 in PBS, Cell Signaling), overnight, at 4°C. For all antibodies, a negative control was performed by replacing the primary antibody with a class-matched immunoglobulin. Slides were labeled by the streptavidin-biotin-peroxidase complex technique with a commercial immunoperoxidase kit (LSAB + System-HRP, Dako, Carpinteria, CA, USA). Positive cells became evident after development with DAB (3′3-diaminobenzidine tetrahydrochloride) and hematoxylin as a counterstain. Images were obtained with Image-ProPlus 4.5 software (Media Cybernetics, Silver Spring, MD, USA). Quantitative analysis for BrdU incorporation was undertaken using the proliferation index (PIx), calculated by the relation between the number of BrdU-positive cells per 1000 cells counted for each case studied, showing the results in percentage [IPx = (number of BrdU − positive cells/1000 cells) × 100]. Quantification of caspase-3 positive cells was performed with the same method and expressed as caspase-3 activation index.

### 2.9. DNA Fragmentation Assay

To verify the effects of the SF-100% treatment on DNA integrity, we performed the DNA smear technique. This technique consists of electrophoresis of full genomic extracted DNA resolved in 1.5% agarose gel. 2 × 10^6^ cells/well were plated on 6-well plates and underwent treatment with SF-100%. Cells were washed in PBS and centrifuged for 5 min, at 1200 rpm, 4°C. The pellet was resuspended in a lysis solution (NaCl 0.1 M, Tris-HCl 0.05 M, EDTA 0.1 M, 0.49 mg protease K/mL, and SDS 1%) and incubated for 2 h at 65°C. After 2 h, samples were centrifuged (10000 rpm, 4°C for 20 min) and the supernatant was collected. Ice-cold ethanol (70%, twice the supernatant volume) and ice-cold sodium acetate (3 M, 10% of total volume) were added. This solution was then gently homogenized until DNA formed a cluster and then centrifuged at 6000 rpm, 4°C, for 10 min. Ethanol (500 *μ*L, 70%, 4°C) was used to wash DNA twice and the pellet was dried. Pellets were dissolved in TE buffer (20 *μ*L, 10 mM Tris-HCl, 1 mM EDTA) and were conserved at −20°C until use. Full genomic DNA yield was measured photometrically at 260 nm (Eppendorf). 10 *μ*L of this DNA extract with 5 *μ*L of bromophenol blue was applied in each lane onto a 1.5% agarose gel, a well received molecular weight marker, which indicates the presence of breaks in DNA samples. Electrophoresis was performed in 60 V for 1.5 h. Afterward, the gel was stained with ethidium bromide and analyzed under UV light using the Image Master VDS-CL (Amersham Pharmacia Biotech).

### 2.10. Western Blot Analysis

Western blot analysis was performed under the same real-time PCR treatment times. 2 × 10^7^ cells were plated (100 × 20 mm plates) and underwent SF-100% treatment for 48 and 72 h (*n* = 6/time). After each treatment time, the medium was removed and cells were washed three times with buffer solution. Cultures were lysed in buffer containing 50 mmol/L Tris-HCl, pH 7.4, 1% Nonidet P-40, 0.25% sodium deoxycholate, 150 mmol/L NaCl, 1 mmol/L EDTA, and protease inhibitors cocktail (Sigma, St. Louis, MO, USA). Samples were centrifuged at 16,000 g for 15 min to remove insoluble material and stored at −20°C. Protein concentrations were measured using the Bio-Rad Protein Assay. Thirty-five micrograms of protein was resolved on 13% SDS-polyacrylamide gel and transferred to nitrocellulose membranes (I-blot Dry Blotting System, Invitrogen, Carlsbad, CA, USA). Membranes were probed with rabbit anti-mouse antibodies to CDK2, CDK4, CDK6, cyclin D1, cyclin D3, cyclin E, and p27 (Santa Cruz Biotechnology, Inc., Santa Cruz, California, USA, 1 : 200), overnight, at 4°C. After incubation with peroxidase-labeled secondary antibody bovine anti-rabbit IgG-HRP (Santa Cruz Biotechnology, Inc., Santa Cruz, California, USA, 1 : 2000) the membranes were developed with chemoluminescence system ECL Plus and visualized in a Hyperfilm ECL (GE HealthCare, Piscataway, NJ, USA). Beta-actin (Sigma Aldrich, 1 : 500) was used as endogenous control. The relative density of the immunoreactive bands was measured by densitometry (Image Master System, GE HealthCare, Piscataway, NJ, USA).

### 2.11. Real-Time PCR

Real-time PCR was performed in treatment times which showed the greatest reduction in HepG2 cells development. 2 × 10^6^ cells/well were plated on 6-well plates and underwent SF-100% treatment for 48 and 72 h. After each treatment time, total RNA was extracted from the cell culture with RNAspin Mini RNA Isolation Kit (GE, HealthCare, Piscataway, NJ, USA) and reverse-transcribed to yield cDNA. For reverse transcription (RT), we used Taqman Reverse Transcription Reagents (Applied Biosystems, Foster City, CA, USA). Primers and probes for real-time PCR were purchased from Applied Biosystems, and mRNA expression of CDK2 (Assay ID Hs00608082_m1), CDK4 (Assay ID Hs00262861_m1), CDK6 (Assay ID Hs00608037_m1), p27^KIP1^ (Assay ID Hs00153277_m1), cyclin D1 (Assay ID Hs00277039_m1), cyclin D3 (Assay ID Hs00426901_m1), and cyclin E (Assay ID Hs00233356_m1) was determined with Taqman 20 assays-on-demand expression assay mix (Taqman Universal Master Mix, n°. 4304437; Applied Biosystems). The beta-actin gene (Assay ID Hs00242273_mL) was used as housekeeping gene to normalize the results. Each sample was analyzed in duplicate for each gene, and negative controls were enrolled. The real-time PCR machine was an ABI PRISM 7000 (Applied Biosystems, Foster City, CA, USA). Analysis of relative gene expression data was performed according to the 2^−ΔΔCT^ method [14].

### 2.12. Statistical Analysis

All results shown represented the mean ± SD. Comparison of parameters was made relative to untreated control groups using Mann-Whitney or Student* t*-tests with two-tailed comparisons. A *P* value of less than 0.05 indicated a significant difference between groups.

## 3. Results

### 3.1. The SF-100% from* H. paniculata* Roots Reduced HepG2 Cell Viability

**Figure 1:**
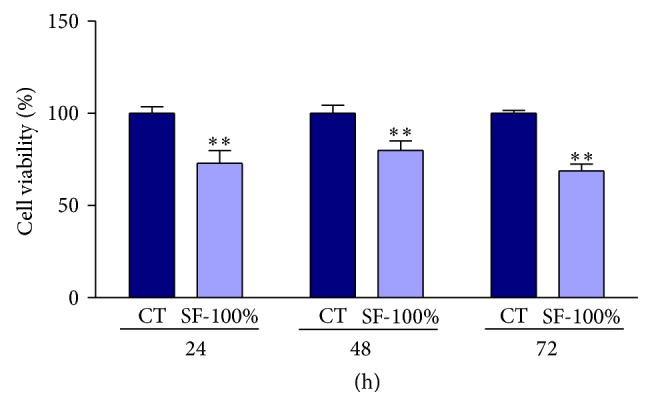
Effect of antitumor activity of pfaffosidic fraction from* Hebanthe paniculata* roots on HepG2 cell growth. The cells were exposed to SF-100% at 100 *μ*g/mL for 24, 48, or 72 h and the viability was evaluated by MTT assay. The treatment decreased cell growth at all times of treatment. The growth was expressed as mean ± SD. Mann-Whitney test, ^∗∗^ *P* < 0.01 in comparison to control.

The MTT assay was employed to evaluate the toxicity of SF-100% on human hepatocellular carcinoma cell line HepG2. Treatment with the pfaffosidic fraction significantly decreased (*P* < 0.001) cell viability from 24 to 72 h in comparison to control (Figure 1). Initially, viability of HepG2 cells decreased 27% after 24 h; however, the reduction was greater at 72 h, with a decrease of 31%.

### 3.2. The SF-100% from* H. paniculata* Roots Increased Sub-G1 Cells Population

**Figure 2:**
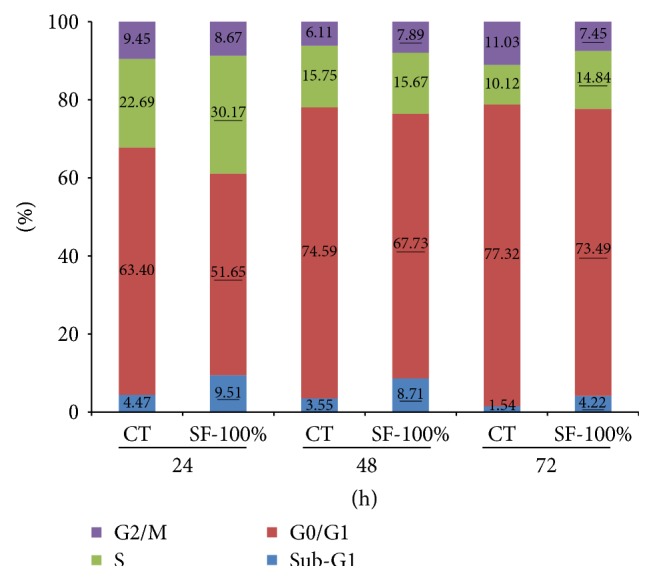
Flow cytometry analysis of cell cycle distribution. The cell cycle was assessed using PI staining. The effects of pfaffosidic fraction from* Hebanthe paniculata* roots on cell cycle progression were determined for 24, 48, or 72 h after treatment. Each value represents the average percentage. The significant differences in comparison to control are underlined. Mann-Whitney test, *P* < 0.05.

To elucidate whether the mechanism of reduction in the cell viability also involves cell cycle changes, the effects of pfaffosidic fraction on cell cycle progression were determined by flow cytometry. The cell cycle showed a variable behavior in different treatment times. It was clear that as incubation time increased; the percentage of cells in the DNA replication phase (S) decreased and consequently increased in the resting or initial phase (G0/G1) independent of the treatment. However, at 24 h incubation time the SF-100% increased cell populations in sub-G1 (dead or haploid cells) and S phases compared to control treatment. This effect on cells in the S phase was not so clear after 48 and 72 h of SF-100% treatment likely due to the incubation time that also affected cells in this phase (Figure 2).

### 3.3. The SF-100% from* H. paniculata* Roots Reduces Cell Proliferation

**Figure 3:**
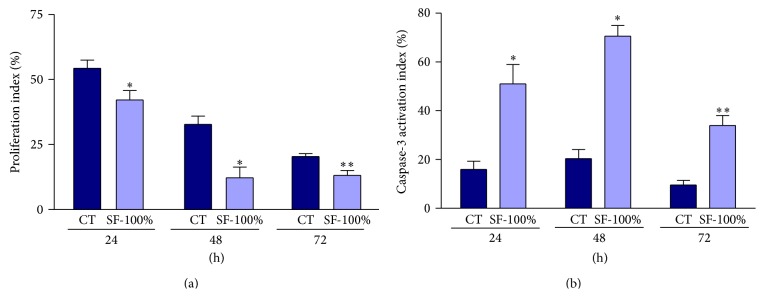
Effects of SF-100% from* Hebanthe paniculata* roots on the HepG2 cell proliferation or caspase-3 activation. These analyses were assessed by measurement of BrdU incorporation and caspase-3 positive cells for 24, 48, or 72 h after treatment. (a) Proliferation index calculated to BrdU incorporation. (b) Caspase-3 activation index. Each value represents the mean ± SD. Mann-Whitney test, ^∗^ *P* < 0.05, ^∗∗^ *P* < 0.01 in comparison to control.

To verify whether SF-100% abrogates DNA replication and delays cell cycle, BrdU was used (analog of thymidine) to identify replicating cells after treatment. BrdU-positive cells were detected by immunocytochemistry and the proliferation index (PIx) was calculated. As observed in cell cycle analysis, the incubation time reduced DNA replicating cells independent of treatment. However, when compared between treatments, the PIx significantly decreased 22% at 24 h, 62% at 48 h, and 36% at 72 h of incubation with SF-100% (Figure 3(a)). These results indicated that the SF-100% reduced proliferating cells in all experimental periods.

### 3.4. The SF-100% from* H. paniculata* Roots Induces Apoptosis by Caspase-3 Activation

To determine whether treatment with pfaffosidic fraction increased sub-G1 cell population by inducing apoptosis, caspase-3 positive cells were measured by immunocytochemistry and the caspase-3 activation index was calculated. As shown in Figure 3(b) the index increased 3-fold at 24 h (51.03 versus 15.95%) and 3.5-fold at 48 h (70.58 versus 20.35%) and 72 h (33.96 versus 9.58%) in comparison with control cells. These results indicate that SF-100% increased sub-G1 cell population inducing apoptosis via caspase-3 activation.

### 3.5. The SF-100% from* H. paniculata* Roots Does Not Affect DNA Integrity

**Figure 4:**
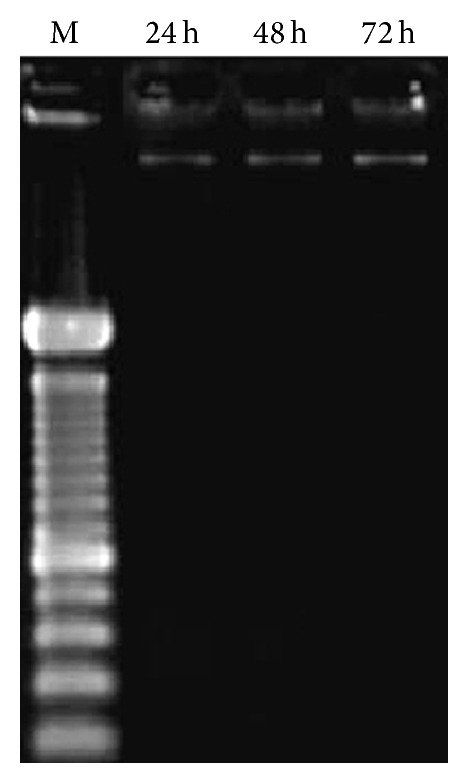
Electrophoretic examination of the DNA fragment of HepG2 cells. DNA was extracted from HepG2 cells at 24, 48, or 72 h after treatment with SF-100% from* Hebanthe paniculata* roots. DNA was separated on a 1.5% agarose gel. M is the 100 bp DNA marker.

We also analyzed whether the apoptosis induction shown by HepG2 cells after the pfaffosidic fraction treatment was a result of DNA strand breaks. This hypothesis was investigated by analysis of DNA fragmentation using a classical DNA laddering on agarose gel electrophoresis. The treatment did not affect DNA integrity (Figure 4).

### 3.6. The SF-100% from* H. paniculata* Roots Reduces Cyclin D1 and Cyclin D3 and Increased p27^KIP1^ Protein and Gene Expression

**Figure 5:**
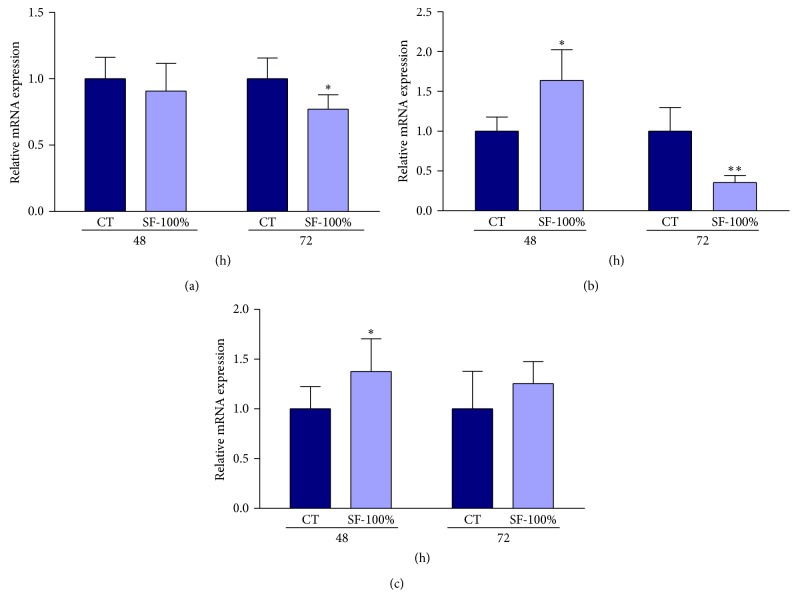
Effect of SF-100% on mRNA expression of cell cycle-related genes for 48 or 72 h after treatment. The gene expression relative of CDK2, -4, and -6, cyclins D1, D3, and E, and p27^KIP1^ were detected by real-time PCR. Only significant changes in expression were shown here. (a) Cyclin D1. (b) Cyclin D3. (c) p27^KIP1^. Mann-Whitney test, ^∗^ *P* < 0.05, ^∗∗^ *P* < 0.01 in comparison to control.

**Figure 6:**
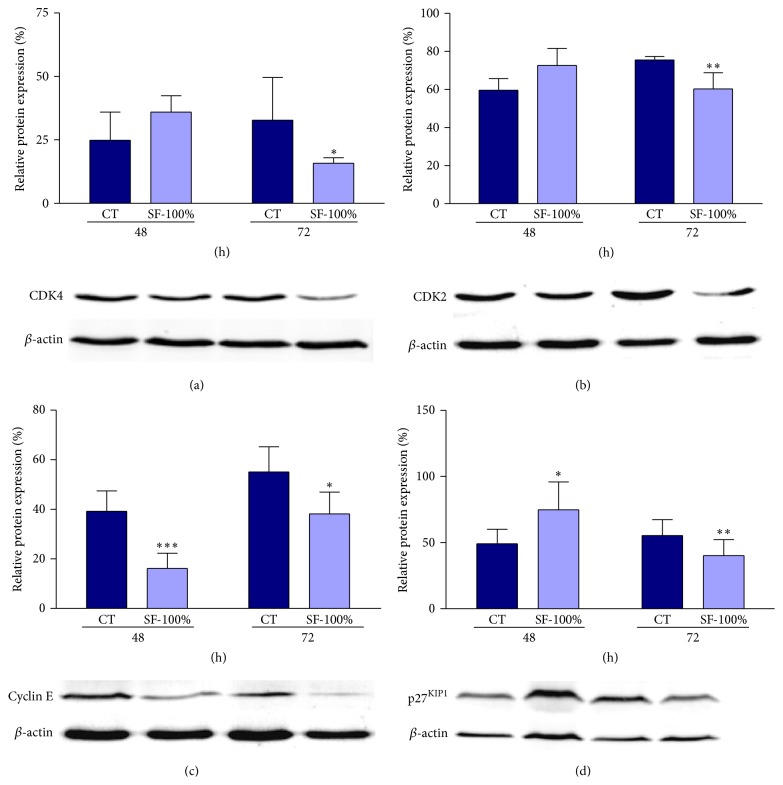
Effect of SF-100% on expression of cell cycle-related proteins for 48 or 72 h after treatment. Levels of CDK2, -4, and 6-, cyclins D1, D3, and E, and p27^KIP1^ were detected by Western blot. Only significant changes in expression were shown here. (a) CDK4. (b) CDK2. (c) Cyclin E. (d) p27^KIP1^. Mann-Whitney test, ^∗^ *P* < 0.05, ^∗∗^ *P* < 0.01, and ^∗∗∗^ *P* < 0.001 in comparison to control.

To understand the mechanism of SF-100% induced cell growth inhibition and apoptosis, the expression of the cell cycle-related proteins CDK2, -4, and -6, cyclins D1, D3, and E, and p27^KIP1^ was investigated by quantitative PCR analysis (Figure 5) and Western blot (Figure 6) at 48 or 72 h after treatment. SF-100% treatment significantly increased and decreased cyclin D3 gene expression in the same proportion (64%, Figure 5(b)) during the analysis time. An expression reduction was observed in cyclin D1 gene at 72 h (23%, Figure 5(a)) and p27^KIP1^ was upregulated only at 48 h after treatment (38%, Figure 5(c)). As shown in Figures 6(a)–6(d), a significant reduction was observed in CDK4 (52%, Figure 6(a)) and CDK2 (20%, Figure 6(b)) expression at 72 h. SF-100% treatment reduced cyclin E expression in all experimental periods (59% and 31%, resp., Figure 6(c)) and upregulated p27^KIP1^ (52%, Figure 6(d)) at 48 h; however, this increase was not maintained up to 72 h (28%, Figure 6(d)). The other genes and proteins showed no significant differences (data not shown).

## 4. Discussion

Roots from* Hebanthe paniculata* (formerly* Pfaffia paniculata* and popularly known as Brazilian ginseng) and its extracts showed antiproliferative properties in several experimental models* in vivo* and* in vitro*. In fact, previous studies from our group showed that diet containing 2% of* H. paniculata* powdered root (2% PP) has a chemopreventive effect in an infant mouse hepatocarcinogenesis model induced by diethylnitrosamine (DEN) [5, 6]. In the last study, it was noted that this effect could be a result of increased apoptotic cell death and reduced proliferation, since PCNA-positive hepatocyte number was lower in mice treated with 2% PP diet. Additionally, it was demonstrated by our group that butanolic extract of this root (contain pfaffosides A–F) was responsible for the antitumoral effect, given that it increased survival of Ehrlich tumor-bearing mice [7]. Nonetheless, the mechanism of action of pfaffosides, which are the main active principles in this root, is not completely understood. Thus, in this study we investigated the effect of purified pfaffosidic fraction from* H. paniculata* on survival and proliferation of a human hepatocellular carcinoma cell line (HepG2) and found that pfaffosides inhibit proliferation and induce apoptosis of HepG2 cells.

Pfaffosides are triterpenoid saponins found in* H. paniculata* root that are associated with its therapeutic properties [10]. Studies with pfaffosides A–F demonstrated cytotoxicity and inhibitory effects on the growth of cells from murine B-16 melanoma [9, 10, 15]. Apparently, the capacity to inhibit tumor growth is a property observed in the saponin group, given that saponins extracted from different plants also cause growth inhibition of different tumor cell lines [16–20], as well as HepG2 cells [21–24]. According to Fuchs et al. [25], antitumorigenic effects are the most interesting properties of the saponins, mainly inhibiting tumor cell growth by cell cycle arrest and apoptosis. Indeed, we observed that pfaffosidic fraction reduced cell viability and induced cell cycle changes in HepG2 cells such as S phase arrest and increase in the sub-G1 cell population that were more evident after 24 h of treatment.

It is well known that cell cycle progression is tightly regulated by the activity of protein kinases complexes, each consisting of a cyclin-dependent kinase (CDK) and cyclins. Cyclin/CDK complexes are formed and activated to control the entry into and passage through various phases of cell cycle [26]. Progression through G1 is regulated by cyclin D/CDK4 or CDK6, while cyclin E/CDK2 is required for the G1/S transition. Cyclin A/CDK2 plays a critical role in the control of S phase and is also essential for G2 progression. Cyclin A- and cyclin B-associated CDK1 (CDC2) regulates the G2/M phases. CDK inhibitors such as p27^KIP1^, p21^CIP1^, and p57^KIP2^ are responsible for negatively regulated cell cycle, so in quiescent cells p27^KIP1^ levels are high, but once cells enter the cycle, they fall [26]. These inhibitory proteins bind all cyclin/CDK complexes and inhibit their activity by fitting into the ATP binding site of CDKs but, when highly expressed, impair also the activating phosphorylation mediated by the CAK complex (CDK7/cyclin H/Mat1) [27]. p21 is transcriptionally controlled by tumor suppressor protein p53 and appears to be required for G1 phase arrest following DNA damage [28]. p27 is primarily regulated by ubiquitin-dependent proteolysis and its protein level is high in early- and mid-G1 to prevent untimed activation of cyclin E/cyclin A-CDK2 to progress into the S phase, but at the late G1 or in S phase the p27^KIP1^ protein is targeted for degradation through its phosphorylation at threonine 187 (T187) by kinases such as cyclin E/CDK2 or cyclin A/CDK2 [29, 30]. Consistent with this notion, here we demonstrated that treatment of HepG2 cells for 48 h with purified pfaffosidic fraction inhibits gene expression of cyclin E and increases gene expression of cyclin D3 and increases both gene expression and protein levels of p27^KIP1^. Considering that high levels of p27^KIP1^ can cause S phase arrest by preventing activation of cyclin E/CDK2, this could be the explanation by which HepG2 cells undergo cell cycle arrest in the S phase during pfaffosidic exposure. In accordance with our results, a study evaluated the effects of Gatifloxacin, a new antibiotic, under pancreatic cell line Panc-1. The authors reported that Gatifloxacin showed anticancer activity in the pancreatic cell line by S phase cell cycle arrest, due to increased levels of p27^KIP1^ [31]. However, Gatifloxacin did not induce apoptosis of Panc-1 cell.

After 72 h of treatment of HepG2 cells with pfaffosidic fraction, we also observed reduction in protein levels of cyclin E, CDK2, CDK4, and p27^KIP1^ and in gene expression of cyclin D1 and cyclin D3. Taking into account that cyclin D/CDK4 is required for progression through G1 phase and cyclin E/CDK2 is required for the G1/S transition [26], we could hypothesize that the inhibition of these cell cycle proteins be responsible for time-dependent inhibition of DNA replication in HepG2 cells as clearly demonstrated by lower percentage of BrdU-positive cells after treatment with pfaffosidic fraction. Additionally, the treatment with pfaffosidic fraction induced a sub-G1 peak suggesting that it induces apoptotic cell death in HepG2 cells. In fact, a study showed that depletion of cyclin E by siRNA in HCC cell lines (Hep3B and HepG2), which are cyclin E-overexpressed cells, was responsible for inducing growth arrest and apoptosis [32]. Taken together, inhibition of both gene transcription (mRNA) and protein expression of cyclin E by pfaffosidic fraction from* P. paniculata* could be considered the mechanism of its antitumor activity. Additional evidence for proliferation inhibition and apoptosis induction by depletion of cyclin E was reported in primary cells obtained from DEN-induced mouse liver tumors where cyclin E silencing allows p53* de novo* expression and activity inducing p21 and reducing antiapoptotic Bcl-XL levels [33]. This study corroborates our hypothesis that cyclin E depletion is responsible for antitumor activity of* H. paniculata* since previous results from our group using the same mouse hepatocarcinogenesis model induced by diethylnitrosamine (DEN) also evidenced that* H. paniculata* powdered root reduced proliferation and increased apoptotic cell death [6].

Apoptosis is a highly controlled process of programmed cell death, responsible for the elimination of undesirable and defective cells, and it is one of the mechanisms of action found in antitumoral drugs [34]. The caspase family plays an important role in the regulation of apoptosis. Caspase activation is a hallmark of apoptosis induction in response to death-inducing signals originating from cell-surface receptors, mitochondria, or the endoplasmic reticulum [35]. In particular, activation of caspase-3 plays a central role in the initiation of apoptosis [36]. As an effector caspase, the death-receptor and mitochondrial pathways converge at the level of caspase-3 activation. The mitochondrial pathway is used extensively in response to extracellular stimulus and internal insults such as DNA damage to induce apoptosis [37]. In the present study, we further analyzed the induction of apoptotic cell death through caspase-3 activation and confirmed that pfaffosidic fraction induces HepG2 cell apoptosis by caspase-3 activation in a time-independent manner. However, the activation of caspase-3 in HepG2 cells was not a consequence of DNA fragmentation being probably just a consequence of cyclin E deregulation.

In conclusion, results demonstrated that antitumoral effects of pfaffosidic fraction obtained from* H. paniculata* roots were due to S phase cell cycle arrest and induction of apoptosis by activation of caspase-3. The increasing knowledge of the antitumor cell cycle based properties of* H. paniculata* roots can provide the development of new antineoplastic therapies for the hepatocellular carcinoma since these cells proliferate at high rates compared to normal hepatocytes that are mostly quiescent.

## Abbreviations

BrdU: 5-Bromo-2-deoxyuridine
CDK: Cyclin-dependent kinase
CDKI: CDK inhibitors
DMEM: Dulbecco's modified Eagle medium
DMSO: Dimethyl sulfoxide
EtOH: Ethanol
HCC: Hepatocellular carcinoma
MeOH: Methanol
mRNA: Messenger RNA
MTT: 3-(4.5-Dimethylthiazol-2-yl)-2.5-diphenyltetrazolium bromide
PBS: Phosphate-buffered saline
PI: Propidium iodide
Pix: Proliferation index
RT: Room temperature
RT-PCR: Real-time reverse transcription-polymerase chain reaction
SDS: Sodium dodecyl sulphate
SF: Saponinic fraction
TE: Tris/EDTA.

## References

[1] Parkin D. M., Bray F., Ferlay J., Pisani P. (2005). Global cancer statistics, 2002. *CA: A Cancer Journal for Clinicians*.

[2] Wu P., Dugoua J. J., Eyawo O., Mills E. J. (2009). Traditional Chinese medicines in the treatment of hepatocellular cancers: a systematic review and meta-analysis. *Journal of Experimental & Clinical Cancer Research*.

[3] Frau M., Biasi F., Feo F., Pascale R. M. (2010). Prognostic markers and putative therapeutic targets for hepatocellular carcinoma. *Molecular Aspects of Medicine*.

[4] Matsuzaki P., Akisue G., Oloris S. C. S., Górniak S. L., Dagli M. L. Z. (2003). Effect of *Pfaffia paniculata* (Brazilian ginseng) on the Ehrlich tumor in its ascitic form. *Life Sciences*.

[5] da Silva T. C., da Silva A. P., Akisue G. (2005). Inhibitory effects of *Pfaffia paniculata* (Brazilian ginseng) on preneoplastic and neoplastic lesions in a mouse hepatocarcinogenesis model. *Cancer Letters*.

[6] da Silva T. C., Cogliati B., da Silva A. P. (2010). Pfaffia paniculata (Brazilian ginseng) roots decrease proliferation and increase apoptosis but do not affect cell communication in murine hepatocarcinogenesis. *Experimental and Toxicologic Pathology*.

[7] Matsuzaki P., Haraguchi M., Akisue G. (2006). Antineoplastic effects of butanolic residue of *Pfaffia paniculata*. *Cancer Letters*.

[8] Nagamine M. K., da Silva T. C., Matsuzaki P. (2009). Cytotoxic effects of butanolic extract from *Pfaffia paniculata* (Brazilian Ginseng) on cultured human breast cancer cell line MCF-7. *Experimental and Toxicologic Pathology*.

[9] Nishimoto N., Nakai S., Takagi N. (1984). Pfaffosides and nortriterpenoid saponins from *Pfaffia paniculata*. *Phytochemistry*.

[10] Schenkel E. P., Gosman G., Athayde M. L. S., medicamento ao. (2007). *Farmacognosia da planta ao medicamento*.

[11] Nakai S., Takagi N., Miichi H. (1984). Pfaffosides, nortriterpenoid saponins, from *Pfaffia paniculata*. *Phytochemistry*.

[12] Haraguchi M., Mimaki Y., Motidome M. (2000). Steroidal saponins from the leaves of *Cestrum sendtenerianum*. *Phytochemistry*.

[13] Santos F. M., Latorre A. O., Hueza I. M. (2011). Increased antitumor efficacy by the combined administration of swainsonine and cisplatin in vivo. *Phytomedicine*.

[14] Livak K. J., Schmittgen T. D. (2001). Analysis of relative gene expression data using real-time quantitative PCR and the 2^−ΔΔCT^ method. *Methods*.

[15] Takemoto T., Nishimoto N., Nakai S. (1983). Pfaffic acid, a novel nortriterpene from *Pfaffia paniculata* Kuntze. *Tetrahedron Letters*.

[16] Chen L., Chen J., Xu H. (2013). Sasanquasaponin from Camellia oleifera Abel. induces cell cycle arrest and apoptosis in human breast cancer MCF-7 cells. *Fitoterapia*.

[17] Tang X. P., Tang G. D., Fang C. Y., Liang Z. H., Zhang L. Y. (2013). Effects of ginsenoside Rh2 on growth and migration of pancreatic cancer cells. *World Journal of Gastroenterology*.

[18] Li Q., Li W., Hui L.-P., Zhao C.-Y., He L., Koike K. (2012). 13,28-Epoxy triterpenoid saponins from *Ardisia japonica* selectively inhibit proliferation of liver cancer cells without affecting normal liver cells. *Bioorganic and Medicinal Chemistry Letters*.

[19] Kim S. M., Lee S. Y., Cho J. S. (2010). Combination of ginsenoside Rg3 with docetaxel enhances the susceptibility of prostate cancer cells via inhibition of NF-*κ*B. *European Journal of Pharmacology*.

[20] Lee W.-H., Choi J.-S., Kim H. Y., Park J.-H., Lee S.-K., Surh Y.-J. (2009). Heat-processed neoginseng, KG-135, down-regulates G1 Cyclin-dependent kinase through the proteasome-mediatedpathway in HeLa cell. *Oncology Reports*.

[21] Li Q., Deng L., Li W., Koike K. (2014). Cyclamin, a natural 13,28-epoxy triterpenoid saponin, synergistically enhances the cytotoxicity of chemotherapeutic drugs in human liver cancer cells but not non-neoplastic liver cells. *Planta Medica*.

[22] Qin H., Du X., Zhang Y., Wang R. (2014). Platycodin D, a triterpenoid saponin from *Platycodon grandiflorum*, inducesG2/M arrest and apoptosis in human hepatoma HepG2 cells by modulating the PI3K/Akt pathway. *Tumor Biology*.

[23] Jang S.-I., Lee Y.-W., Cho C.-K., Yoo H.-S., Jang J.-H. (2013). Identification of target genes involved in the antiproliferative effect of enzyme-modified ginseng extract in HepG2 hepatocarcinoma cell. *Evidence-Based Complementary and Alternative Medicine*.

[24] Wang Y., Deng L., Zhong H., Jiang X., Chen J. (2011). Natural plant extract tubeimoside I promotes apoptosis-mediated cell death in cultured human hepatoma (HepG2) cells. *Biological and Pharmaceutical Bulletin*.

[25] Fuchs H., Bachran D., Panjideh H. (2009). Saponins as tool for improved targeted tumor therapies. *Current Drug Targets*.

[26] Sherr C. J. (1996). Cancer cell cycles. *Science*.

[27] Bisteau X., Caldez M. J., Kaldis P. (2014). The complex relationship between liver cancer and the cell cycle: a story of multiple regulations. *Cancers (Basel)*.

[28] El-Deiry W. S. (1997). Regulation of p53 downstream genes. *Seminars in Cancer Biology*.

[29] Montagnoli A., Fiore F., Eytan E. (1999). Ubiquitination of p27 is regulated by Cdk-dependent phosphorylation and trimeric complex formation. *Genes & Development*.

[30] Sheaff R. J., Groudine M., Gordon M., Roberts J. M., Clurman B. E. (1997). Cyclin E-CDK2 is a regulator of p27Kip1. *Genes and Development*.

[31] Yadav V., Sultana S., Yadav J., Saini N. (2012). Gatifloxacin induces S and G2-phase cell cycle arrest in pancreatic cancer cells via p21/p27/p53. *PLoS ONE*.

[32] Li K., Lin S.-Y., Brunicardi F. C., Seu P. (2003). Use of RNA interference to target cyclin E-overexpressing hepatocellular carcinoma. *Cancer Research*.

[33] Pok S., Wen V., Shackel N. (2013). Cyclin E facilitates dysplastic hepatocytes to bypass G1/S checkpoint in hepatocarcinogenesis. *Journal of Gastroenterology and Hepatology*.

[34] Park H.-J., Choi S. Y., Hong S. M., Hwang S. G., Park D. K. (2010). The ethyl acetate extract of *Phellinus linteus* grown on germinated brown rice induces G_0_/G_1_ cell cycle arrest and apoptosis in human colon carcinoma HT29 cells. *Phytotherapy Research*.

[35] Yang L., Wu S., Zhang Q., Liu F., Wu P. (2007). 23,24-Dihydrocucurbitacin B induces G2/M cell-cycle arrest and mitochondria-dependent apoptosis in human breast cancer cells (Bcap37). *Cancer Letters*.

[36] Nicholson D. W., Ali A., Thornberry N. A. (1995). Identification and inhibition of the ICE/CED-3 protease necessary for mammalian apoptosis. *Nature*.

[37] Hengartner M. O. (2000). The biochemistry of apoptosis. *Nature*.

